# Microstructure and Mechanical Properties of AlSi10MnMg Alloy with Increased Content of Recycled Scrap

**DOI:** 10.3390/ma18051119

**Published:** 2025-03-01

**Authors:** Jaroslaw Piatkowski, Katarzyna Nowinska, Tomasz Matula, Grzegorz Siwiec, Michal Szucki, Beata Oleksiak

**Affiliations:** 1 Department of Material Technologies, Faculty of Materials Science, Silesian University of Technology, Krasinskiego 8, 40-019 Katowice, Poland; jaroslaw.piatkowski@polsl.pl; 2 Department of Electrical Engineering and Automation in Industry, Faculty of Mining, Safety Engineering and Industrial Automation, Silesian University of Technology, ul. Akademicka 2, 41-100 Gliwice, Poland; 3 Department of Metallurgy and Recycling, Faculty of Materials Science, Silesian University of Technology, Krasinskiego 8, 40-019 Katowice, Poland; tomasz.matula@polsl.pl (T.M.); grzegorz.siwiec@polsl.pl (G.S.); 4 Foundry Institute, Technische Universität Bergakademie Freiberg, 4 Bernhard-von-Cotta-Str., 09599 Freiberg, Germany; michal.szucki@gi.tu-freiberg.de; 5 Department of Production Engineering, Faculty of Materials Science, Silesian University of Technology, Krasinskiego 8, 40-019 Katowice, Poland; beata.oleksiak@polsl.pl

**Keywords:** AlSiMnMg alloys, microstructure, mechanical properties, recycled scrap, porosity, iron content

## Abstract

Increasing the share of circulating scrap in produced castings is not only due to optimizing production costs, but also the need to protect the environment realized by reducing production energy intensity, generating less waste, mitigating greenhouse gas emissions, and consuming fewer natural resources. However, this is associated with maintaining the required properties of castings and considering the impact of impurities on the formation of the structure of aluminum alloys. This research concerns the AlSi10MnMg alloy, which introduces 50 to 75% (every 5%) of circulating scrap. This alloy is one of the most commonly used for producing gravity and pressure die-castings (HPDC), including engine parts and transport structural elements. Based on microscopic research, it was found that the increase in scrap content causes an increase in the share of iron, which results in pre-eutectic (from about 0.45 wt.% to 0.7 wt.% Fe) or even primary crystallization of iron phases (over 0.7 wt.% Fe), mainly the plate–needle phase β-Al_5_FeSi. Its unfavorable morphology and size cause the formation of numerous shrinkage porosity areas, which has an impact on the reduction in mechanical properties (reduction in UTS and YS by approx. 16% and elongation by approx. 18%, compared to the AlSi10MnMg alloy with 50% scrap content). It was found that the increase in the share of recycled scrap (from 50 to 75%) can be used only when the manganese content is increased. Its effect is to change the morphology of the β-Al_5_FeSi phase into α-Al_15_(Fe,Mn)_3_Si_2_, whose crystallization occurs in the temperature range of 540 to 555 °C and increases slightly with increasing manganese addition. It is essential to consider the appropriate value of the Mn/Fe quotient, which should be about 1/2, because a higher value may cause the formation of a sludge factor. This work aimed to determine the limiting iron content (contained in the scrap) at which the sequence of the β-Al_5_FeSi phase release (pre-eutectic or primary crystallization) changes. This sequence mainly affects the form of morphology, the dimensions of the β-Fe phase, and the proportion of shrinkage porosity.

## 1. Introduction

In recent decades, the use of aluminum alloys as a structural material has increased significantly in various areas of industry. Depending on the chemical composition, phase composition, and casting method, a wide range of possible properties of the alloys determine the extent of their usefulness. The use of aluminum alloys as a substitute for heavier (denser) materials is widespread mainly because of the improvement in sustainability and the reduction in CO_2_ during their production processes. With the increasing importance of reducing the weight of castings, such as transportation, among a wide variety of engineering plastics, castable Al-Si-Me (Me = Mg; Ni; Mn; Ti) alloys with peri-eutectic silicon content have gained considerable attention due to their low shrinkage and good flowability.

Among this group of siluminums, one of the most widely used alloys for the production of powertrain castings (e.g., internal combustion engine head covers) [1,2] or vehicle body shell components, so-called “structural” details (e.g., front shock absorbers, door frames, rear stringers, and battery housings for electric cars) is the AlSi10MnMg high-pressure casting (HPDC) alloy [3,4]. The extensive application of this alloy is mainly due to its good mechanical properties (UTS about 300 MPa), especially plastic properties (YS about 100–150 MPa; elongation about 12%) after heat treatment to the T6 or T7 state [5,6], its ability to absorb energy [7], and its ability to be bonded and welded [8,9].

These properties are possible with primary raw materials of high metallurgical purity, whose resources are rapidly dwindling. Therefore, alternative sources of feedstock materials are being sought, for example, from an increased proportion of circulating scrap (post-production and post-depreciation). Additional factors in the use of secondary materials are economic considerations (less energy input for remelting scrap than smelting pure aluminum from bauxite) and environmental requirements—environmental pollution during electrolytic refining of aluminum.

Research in this area has been the subject of many papers, such as [10,11,12], which address many aspects of the increasing proportion of scrap metal and the impact of the impurities it contains and remelting parameters on the physicochemical properties of aluminum alloys. One study [13] found that remelting the metal requires only 5% of the energy needed to produce new aluminum from bauxite ore. Studies [14] indicate that smelting aluminum from ores (so-called primary synthesis) consumes 45 kWh·kg^−1^ due to the high enthalpy of its oxide and about 12 kg CO_2_·kg^−1^, while remelting scrap aluminum (secondary synthesis) consumes only 2.8 kWh·kg^−1^ due to its low melting point (660 °C) and about 0.6 kg CO_2_·kg^−1^ [15]. These data indicate that aluminum recycling has significant potential in balancing energy and reducing the so-called “carbon footprint”, thus leading toward much greater sustainability [16,17], so research in this area is fully justified.

However, it should be noted that the problem of using circulating aluminum scrap is broad and involves many aspects, including its preparation [18], sorting [11], and melting methods [19]. In addition, there are many research results on the effects of gaseous impurities on mechanical properties, surface change (deformation) [20], and corrosion [21].

However, increasing the proportion of scrap in the charge causes the risk of various metallic impurities entering the Al-Si alloy, the worst of which is iron [11,19]. There are few studies on the effect of iron, or rather, of the iron phases emitted, on the kinetics of chemical reactions and the associated changes in the microstructure. As is known, iron most often crystallizes in the form of four phases with different morphologies:

1. β-Al_5_FeSi, also referred to as Al_9_Fe_2_Si in Group 6XX.X (Al-Mg-Si) alloys [22];
2. α-Al_8_Fe_2_Si (possibly α-Al_12_Fe_3_Si_2_), which has a hexagonal structure under thermodynamic equilibrium conditions and only stable in Al-Si-Fe alloys with a very high purity of charge components [23];
3. δ-Al_4_FeSi_2_ present in Al-Si alloys with a more than 18 wt.% Si [24];
4. γ-Al_3_FeSi occurring at more than 4% wt. content Fe and more than 16 wt.% Si [25].

The morphology of these phases (usually polygonal-walled or so-called “chinese script”), referred to as α-phase or π-phase, depends mainly on crystallization conditions and chemical composition. Still, the worst is the β-Al_5_FeSi (β-Fe) phase [26] in Al-Si alloys with up to about 0.4 wt.% Fe; this phase is not very harmful, as it crystallizes after the formation of dendrites from the solid solution α(Al) and the eutectic α(Al) + β(Si). In Al-Si-Mg alloys, in addition to the Mg_2_Si phase, there is a high probability of crystallization of the Al_9_Fe_2_Si (single-stranded) and Al_8_FeMg_3_Si_2_ (hexagonal) phases. Furthermore, under conditions of thermodynamic imbalance, metastable phases β-Al_4_FeSi (25.4 wt.% Fe and 25.5 wt.% Si) and β-Al_3_FeSi (33.9 wt.% Fe and 16.9 wt.% Si) are formed [25]. The Mg_2_Si phase precipitates are dissolved after T6 or T7 heat treatment and are finely precipitated in the α(Al) solid solution matrix [27]. In Al-Si-Cu(Fe) alloys, in addition to Al_2_Cu, various phases crystallize: Al_6_FeCu and Al_7_FeCu_2_ included in multicomponent eutectics of the type α(Al) + Al_X_Fe_Y_Cu_Z_ + β(Si), among others [28,29].

The presence of β-Fe phases in Al-Si alloys becomes problematic when the iron content exceeds 0.4 wt.% for gravity castings and about 1.6 wt.% for die castings (iron prevents castings from sticking to pressure molds, increasing their service life and improving their strength properties at high temperatures [30]). This results in the crystallization of β-Fe phases with a lamellar structure (on the surface of an igneous deposit). This structure causes the propagation of microcracks and stress concentration, which increases the brittleness of Al-Si alloy castings, making them more difficult to machine, and reduces their mechanical properties [31,32], especially ductility [33,34].

An additional reason for reducing these properties in Al-Si(Fe) alloys is excessive shrinkage porosity, which, according to studies [35,36], is also caused by the presence of β-Fe phases. The formation of porosity in aluminum alloys is the result of a combination of two processes [37,38]:

- Shrinkage of the metal during cooling of the casting, which causes a vacuum of the liquid phase in the dendrite growth zone of the solid solution α(Al)—Darcy’s law [39];
- Dissolution and segregation of gases (oxides, sulfides, nitrides) in the metal bath occur with varying intensity, the most important being hydrogen [40,41].

Thus, the rate of heat dissipation and the proportion of impurities (metallic and gaseous) determine porosity, which determines their properties. Studies [26,36] show that porosity negatively affects several functional properties, mainly strength, ductility, and fatigue. According to the study reported in [42], an increase in porosity of about 3% in an AlSi5 alloy reduces its tensile strength by about 40%. The same is true for ductility. Porosity also negatively affects alloy flowability [43] and the tightness of castings, which is often a criterion for material selection [38].

To prevent this, various methods are used to neutralize the effects of harmful β-Fe phases. The most popular are diluting the charge with pure aluminum or introducing multiple additives into the alloy, such as manganese, chromium, cobalt [44,45], or strontium [46,47], which change the morphology of the β-Fe phases from lamellar-igneous to a less harmful one, such as dendritic. Among these additives, one of the most commonly used is manganese [48], which causes the transformation of β-Al_5_FeSi into Al_X_(Fe,Mn)_Y_Si_Z_-type phases, from which Al_15_(Fe,Mn)_3_Si_2_ or Al_12_(Fe,Mn)_3_Si_2_ phases usually crystallize. A multicomponent eutectic of the type α(Al) + Al(Fe,Mn)Si + β(Si) containing the Al_19_Fe_4_MnSi_2_ phase, considered isomorphous to Al_20_Fe_5_Si_2_, can also be formed. In these phases, manganese atoms occupy the positions of iron atoms [49]. The type of morphology of the Al_15_(Fe,Mn)_3_Si_2_ phases, as reported in [46,50], varies and depends on the manganese content and the degree of overcooling of the alloy ΔT. At high-value ΔT, α-Al_15_(Fe,Mn)_3_Si_2_ crystallizes in compact polygons; at slower cooling, the so-called “chinese script” forms. However, it should be remembered that the share of alloying additive for reducing the negative effect of β-Fe phases is strictly dependent on the content of iron, the critical content of which in Al-Si alloys is determined by the following relationship [51]:

Fe_crit._ ≈ 0.075 × (wt.% Si) − 0.05% (1)

According to research results [50,52], when the iron content exceeds 0.45 wt.%, the manganese content should equal about half of the iron content. Exceeding the critical iron content in Al-Si alloys results in crystallizing the previously described β-Fe phases with unfavorable lamellar-igneous morphology and in sludge formation. Its proportion is determined by the so-called “sludge factor” (SF) and the Mn/Fe quotient, the values of which vary depending on the chemical composition of the alloy, manufacturing technology, and metallurgical purity of the feedstock components [53,54]:

S_F_ (wt.%) = Fe+2 × Mn + 3 × Cr (2)

Unfortunately, these views are often theoretical and lack direct experimental evidence; hence, verifying them on commercial Al-Si alloys used for specific castings is necessary. In addition, it has not always been reported how increasing the proportion of circulating scrap and, therefore, increasing the iron content (iron phases) affects some specific mechanical properties and porosity, which are the main criteria determining the sale of Al-Si alloy products.

The purpose of this research was to study the effect of increasing the proportion of circulating scrap (from 50 to 75%, in increments of 5% by weight of the batch) on the mechanical properties and microstructure of the AlSi10MnMg alloy in terms of iron content and the addition of manganese, as a so-called “morphological corrector” of the iron phases.

**Figure 1:**
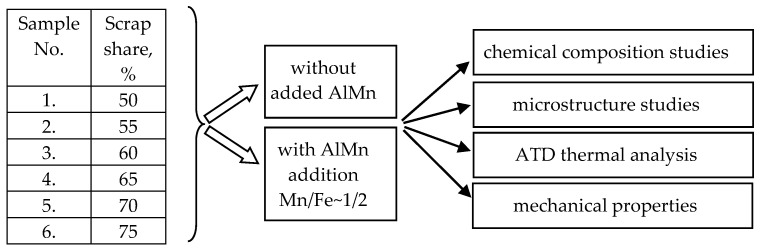
Research plan for the AlSi10MnMg alloy with an increasing share of recycled scrap.

To achieve the stated goal, the scope of this research included the following aspects:

- Perform smelting of AlSi10MnMg alloy with an increasing proportion of circulating scrap according to the experimental plan (Figure 1);
- Investigate the chemical composition of AlSi10MnMg alloy with different iron and manganese contents;
- Analyze the crystallization process by the ATD method for AlSi10MnMg alloy with different iron and manganese contents;
- Test the mechanical properties, i.e., hardness (HB), tensile strength (UTS), conventional yield strength (YS), and elongation (A), based on the static tensile test;
- Characterize the microstructure (including porosity) of the tested alloys.

The goal, therefore, is to determine the limiting iron content (contained in the scrap) at which the sequence of the β-Al_5_FeSi phase release (pre-eutectic or primary crystallization) changes. This sequence mainly affects the form of morphology, the dimensions of the β-Fe phase, and the proportion of shrinkage porosity. These parameters determine the mechanical and ductile properties of the AlSi10MnMg(Fe) alloy and, therefore, its potential applications.

## 2. Materials and Methods

### 2.1. Method of Preparing Research Material

AlSi10MnMg alloy was chosen for the study (due to its numerous applications for gravity and pressure castings), which was smelted from AlSi20 alloy diluted with pure aluminum (99.95 wt.% Al) to obtain a content of about 10 wt.% Si and master alloys: AlMg10 (10 wt.% Mg) and AlMn20 (20 wt.% Mn). According to the literature review, the manganese content was selected to be half that of iron. AlSiFe scrap was added to each melt in an amount of 50 to 75% by weight of the charge. The rest was pure AlSi10MnMg alloy: 9.5–11.0% Si; 0.3–0.6% Fe; 0.4–0.5% Mn; 0.4–0.6% Mg, 0.05–0.08% Ti; other elements and impurities up to 0.05%, and the rest aluminum (wt.%) according to EN 1706:2010 [55]. The feedstock components were melted in a VSG 02/631 Balzers electric furnace (Seco Warwick, Bedburg-Hau, Germany) in a 0.8 L SiC crucible. After withstanding in the furnace, the alloys were modified with AlSr10 master alloy (10 wt.% Sr) and refined with Rafglin-3 in an amount of about 0.05 wt.%. After reaching a temperature of about 740 °C, they were cast into a QC4080 (Heraeus Electronite, Breda, The Netherlands)tester for thermal testing (recording the cooling curve) and into a metal mold for static tensile testing. The experimental plan is shown in Figure 1.

### 2.2. Method of Performing ATD and Chemical Composition Tests

Thermal tests were carried out using the ATD (thermal derivative analysis) method on a bench equipped with a Crystaldigraph NT3-8K multi-channel temperature logger (ZTech-Jura, Gliwice, Poland) using Mlab2 (version 2) cooling curve recording software. The transmitter meets the requirements of EN 61010 and EN 60584 for temperature measurements with thermocouples. The cooling curve T = f(τ) and its first derivative were recorded after time dT/dτ = f’(τ). Chemical composition tests were performed on the surface of the castings by a QC4080 sampler, which were measured with a Foundry Master CCD 01L00113 WAS-AG emission spectrometer (SpectroLab, Kleve, Germany). Ten samples each were made, discarding the two outliers, and the arithmetic mean, rounded to 2 or 3 decimal places, was determined from the remaining samples.

### 2.3. Methodology for Testing Mechanical Properties

Brinell hardness measurement was performed according to PN-EN ISO 6506-1 on a Zwick ZHF1 (ZwickRoell, Ulm, Austria), with a loading force of 187.5 kg using a steel ball of 2.5 mm diameter at 35 s. The static tensile test at ambient temperature was carried out according to PN-EN ISO 6892-1 on an Instron 3382 (Darmstadt, Germany), using a 20:1 ratio and a constant tensile speed of 5 mm·min^−1^. From this test, the tensile strength (UTS), the conventional yield strength (YS), and the percentage elongation of the specimen after rupture (A) were determined. Ten measurements were taken, discarding the two outliers, and the arithmetic mean was calculated from the remaining measurements. The results obtained for HB, UTS, and YS were rounded to a whole number, while elongation A was rounded to two decimal places.

### 2.4. Method of Conducting Microstructure Tests

Samples were cut from the thermal center of the QC-4080 sampler (Heraeus Electronite, Breda, The Netherlands) for metallographic examination. The specimens were ground and polished with polishing pastes of appropriate granularity, then etched in 1% HF acid. Ten metallographic images were taken each, and those shown are representative images of the alloy’s microstructure under study.

Metallographic scans were taken from the rupture site of the sample for mechanical testing. Microstructure observations were performed on a MeF2 light microscope (Reichert, Leuven, Belgium) (SM) and a Hitachi S-3400N scanning electron microscope (Hitachi High-Technologies, Tokyo, Japan) (SEM) equipped with a Thermo Noran energy dispersive X-ray spectrometer (Thermo Fisher Scientific, Potsdam, Germany) (EDS) and Thermo MagnaRay wavelength dispersive X-ray spectrometer (Thermo Fisher Scientific, Potsdam, Germany) (WDS), and an INCA HKL Nordys II backscattered electron diffraction (EBSD) detector (Hitachi High-Technologies, Tokyo, Japan). Some samples were subjected to deep etching in 5% HF acid solution to better indicate the structural components. X-ray powder diffraction (XRD) analyses were performed with a Philips X’Pert diffractometer (Bridge Tronic Global, Irvine, CA, USA), using a copper anode lamp (λCuKα −1.54178 Ǻ) supplied with 30 mA at 40 kV. Recording was performed using the “step-scan” method with a step of 0.04° and a counting time of 10 s, in the 2θ angle range of 20° to 140°. The gap on the incident beam was 1°, and on the deflected beam, 2°. Soller slits of 0.04 mm were used. Qualitative phase analysis was performed using ICDD mapping. DBWS 9807A software (version 20.8.00) was used in the study, and the Pearson VII function was used to describe the profile of the diffraction lines. Ten diffractograms were taken for each alloy. Quantitative microstructure was evaluated using quantitative metallography and image analysis methods using ImageJ software (version 1.46r) [56]. Binarization was performed using the Otsu method. Binary images of iron phases were subjected to morphological transformations to correctly represent their size and shape. The measurement was performed using the surface method. The presented microstructures are a representative picture of the form of the structural components of the AlSi10MnMg alloy.

## 3. Results and Discussion

### 3.1. Chemical Composition Test Results

Table 1 shows the chemical composition of an AlSi10MnMg alloy with an increasing proportion of circulating scrap before and after introducing a manganese additive. After discarding the two outliers, the results are the arithmetic average of the ten measurements.

Analysis of the chemical composition (Table 1) shows that increasing the proportion of circulating scrap in the AlSi10MnMg alloy, both before and after the addition of manganese, results in an increase in iron content from about 0.32 wt.% (for 50% scrap) to about 0.7 wt.% (for 75% scrap). The content of the other alloying elements did not change significantly. The introduction of manganese additive (in the form of AlMn20 master alloy) increased its proportion from 0.18 to 0.36 wt.%, with no change in the content of the other elements.

Another part of the study analyzed cooling curves (from T_liq._ to T_sol._) using the ATD (thermal derivative analysis) method, recording the exothermic effects of the reactions involved in separating structural components.

### 3.2. ATD Thermal Derivative Analysis Test Results

**Figure 2:**
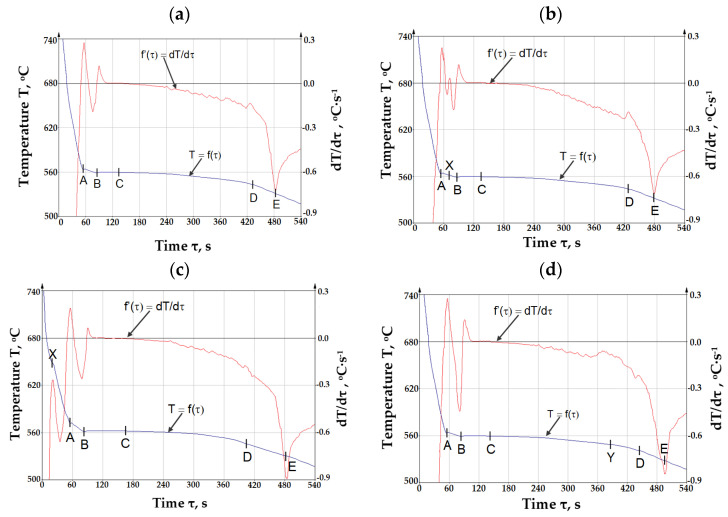
ATD curves of AlSi10MnMg alloy with (**a**) 0.32 (sample No. 1), (**b**) 0.53 (sample No. 4), (**c**) 0.71 (sample No. 6), (**d**) 0.71 wt.% Fe (sample No. 6Mn).

The crystallization curve with the first derivative of AlSi10MnMg alloy with 0.32 (sample No. 1), 0.53 (sample No. 4), 0.71 wt.% Fe (sample No. 6), and after the introduction of manganese (sample No. 6Mn) is shown in Figure 2, while the characteristic temperatures and crystallization process parameters are shown in Table 2.

The symbols in Figure 2 are defined as follows:

- T = f(τ)—the curve of temperature T change at time τ—the so-called TA (temperature analysis) curve;
- dT/dτ = f’(τ)—the first derivative of the temperature change over time—the so-called ATD (thermal derivative analysis) curve;
- Point A—temperature of crystallization of dendrites of solid solution α(Al)—T_α(Al)_ (T_liq._), °C;
- A–B—range of crystallization of dendrites of solid solution α(Al), °C;
- Point B—minimum temperature of crystallization of eutectic α(Al) + β(Si)—T_Emin(α+β)_, °C;
- Point C—average crystallization temperature of the α(Al) + β(Si)—T_E(α+β)_, °C;
- Point X—crystallization temperature of iron intermetallic phases—T_E(Fe)_, °C;
- Point Y—crystallization temperature of iron intermetallic phases—T_E(AlFeMn)_, °C;
- Point D—crystallization temperature of magnesium intermetallic phases—T_E(Mg)_, °C;
- Point E—temperature of the end of crystallization of AlSi10MnMg alloy (T_sol._), °C.

From the data presented in Table 2, it can be seen that increasing the proportion of circulating scrap and thus increasing the iron content in the AlSi10MnMg alloy shifts the formation temperature of the iron phases (T_E(Fe)_) to higher temperatures and changes the order of crystallization of the alloying components. Up to a content of about 0.4 wt.% Fe, crystallization of the iron phases that are likely to be part of the α(Al) + AlFeSi + β(Si) complex eutectic occurs at the same time as the formation of the α(Al) + β(Si) and α(Al) + (MgSi) + β(Si) eutectics. At about 0.4 to about 0.7 wt.%, crystallization of the iron phases becomes pre-eutectic (after the formation of solution dendrites α(Al)), while above 0.7 wt.% Fe, primary crystallization of the iron phases occurs. The effect of the increased proportion of circulating scrap on the remaining crystallization temperatures is negligibly small. The increase in the proportion of circulating scrap is also accompanied by an increase in the range of crystallization (T_liq._ − T_sol._), with an increase in the temperature of T_liq._

### 3.3. Mechanical Properties Test Results

**Figure 3:**
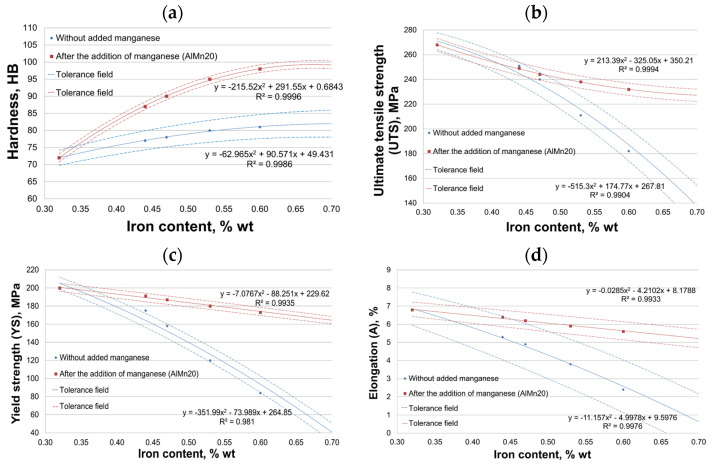
The effect of increasing content of recycled scrap on mechanical properties: (**a**) hardness (HB); (**b**) ultimate tensile strength (UTS); (**c**) yield strength (YS); (**d**) elongation (A) of the AlSi10MnMg alloy before and after the introduction of manganese.

Figure 3 shows the results of mechanical properties: hardness (HB), ultimate tensile strength (UTS), conventional yield strength (YS), and elongation (A) of AlSi10MnMg alloy for increasing circulating scrap content.

Figure 3a shows that the hardness of AlSi10MnMg alloy with increasing scrap percentage increases slightly, from 72 to 82 HB (without manganese addition) and from 72 to 99 HB (after the introduction of manganese addition), so the percentage increase occurred by about 13 and 37%, respectively. Noteworthy are the results of hardness scatter measured by standard deviation. For the alloy without manganese additive, SD(HB) ranges from 4.5 to about 8 HB, while after the introduction of manganese additive, SD decreased to about 2.3 HB. After adding manganese, the smaller scatter of hardness results indicates better homogenization of the AlSi10MnMg alloy structure.

The results in Figure 3b show that the increasing proportion of scrap causes a decrease in tensile strength from 268 to 135 MPa (without manganese) and from 268 to 227 MPa (after the introduction of manganese), that is, by about 50 and 16%, respectively. The downward trend is mainly noticeable after obtaining about 4.5 wt.% Fe. For the alloy without manganese addition, the SD(UTS) ranges from about 14 to about 33 MPa, while it decreased to about 10 MPa after the introduction of manganese. The smaller scatter of UTS results after the addition of manganese may also indicate better homogenization of the structure of the alloy under study.

Figure 3c shows that the increasing proportion of scrap results in a decrease in the conventional yield strength from 200 to 40 MPa (without manganese) and from 200 to 164 MPa (after the introduction of manganese), that is, by about 80 and 18%, respectively. As for UTS, the downward trend is mainly noticeable after obtaining about 4.5 wt.% Fe. SD(YS) ranges from about 14 to about 20 MPa for the alloy without added manganese, while it decreased to about 9 MPa after the introduction. As for HB and UTS, the smaller scatter of SD(YS) results after adding manganese may also indicate better homogenization of the structure of the tested alloy.

The increasing proportion of scrap in the AlSi10MnMg alloy also resulted in a decrease in elongation (Figure 3d) from about 6.8% to about 0.5% (without manganese) and from about 6.8 to 5.2% (after the introduction of manganese), that is, by about 93 and 24%, respectively. There is also a more significant downward trend in elongation after reaching about 4.5 wt.% Fe. The SD(A) of the alloy without manganese ranges from about 1.9 to 3%, and after the introduction of manganese, from about 0.8 to 1%, which may also indicate better homogenization of the structure of the AlSi10MnMg alloy with the addition of manganese.

The changes in the mechanical properties studied should be attributed to the structure of the AlSi10MnMg alloy; hence, the next step was to study its microstructure.

### 3.4. Microstructure Test Results

**Figure 4:**
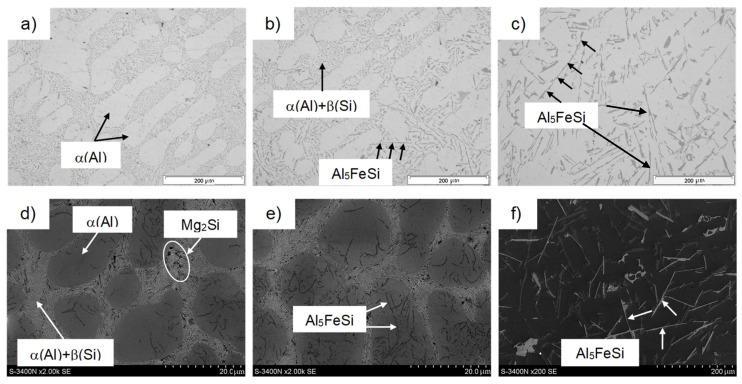
Microstructure of the AlSi10MnMg alloy without manganese addition and with scrap content: (**a**–**d**) approx. 50% (0.32 wt.% Fe); (**b**–**e**) approx. 65% (0.5 wt.% Fe); (**c**–**f**) approx. 70% (0.7 wt.% Fe); (**a**–**c**)—light microscope; (**d**–**f**)—SEM.

**Figure 5:**
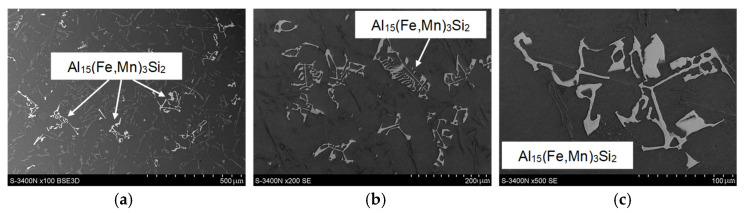
SEM microstructure of AlSi10MnMg alloy with manganese addition: (**a**) 100 times magnification; (**b**) 200 times magnification; (**c**) 500 times magnification.

Figure 4 shows representative microstructures of AlSi10MnMg alloy without adding manganese, and Figure 5 shows them after the introduction of manganese.

**Figure 6:**
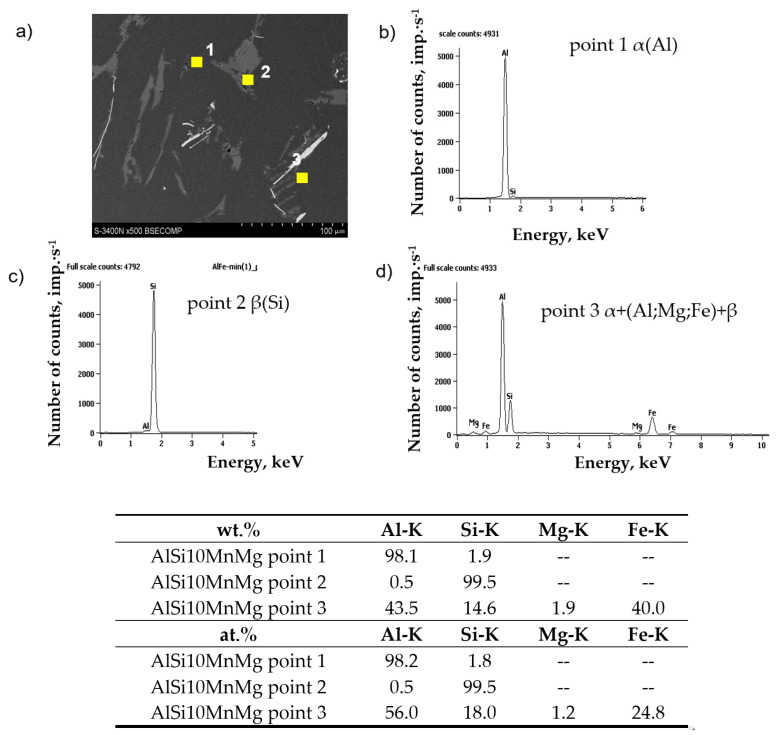
Microstructure (**a**) and results of microanalysis of the chemical composition of AlSi10MnMg alloy without manganese addition in the areas of (**b**) α(Al) matrix; (**c**) silicon crystal β(Si); (**d**) eutectic containing magnesium and iron.

**Figure 7:**
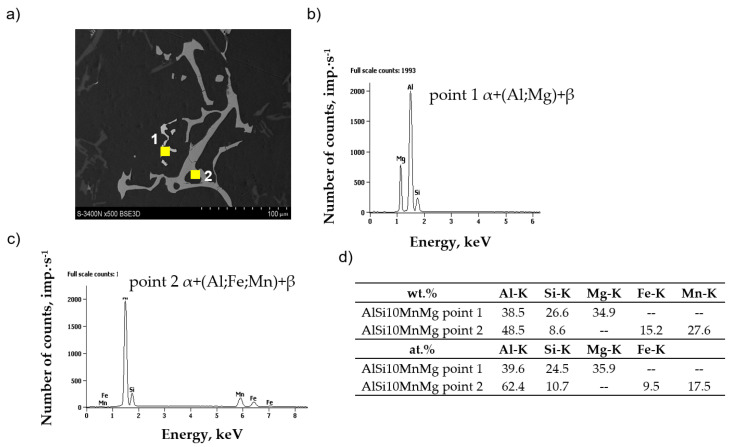
Microstructure (**a**) and results of microanalysis of the chemical composition of the AlSi10MnMg alloy with manganese addition in the areas of the (**b**) eutectic containing magnesium; (**c**) eutectic containing iron and manganese (**d**) chemical composition at points 1 and 2.

**Figure 8:**
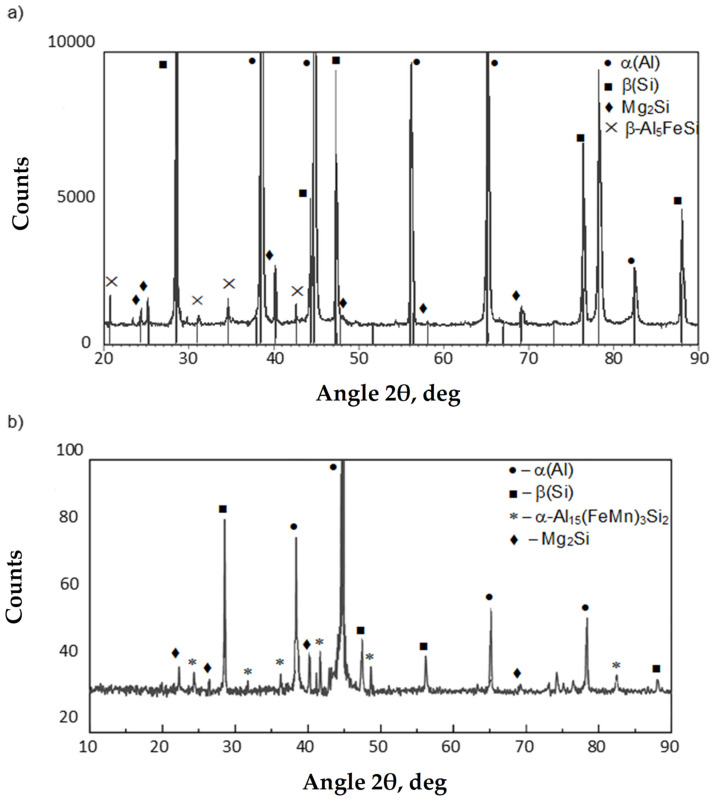
XRD diffraction pattern of the AlSi10MnMg alloy: (**a**) without manganese; (**b**) with manganese addition.

Chemical composition microanalysis and X-ray diffraction studies were performed to identify the components of the AlSi10MnMg alloy structure, shown in Figure 6, Figure 7 and Figure 8.

**Figure 9:**
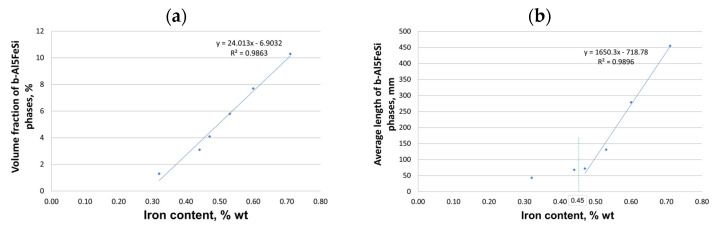
Effect of iron content on (**a**) percentage share; (**b**) average length of β-Al_5_FeSi phases in AlSi10MnMg alloy without manganese addition.

The study of the microstructure of AlSi10MnMg alloy without manganese addition shows that in addition to the traditional components, i.e., dendrites of the solid solution α(Al), silicon crystals included in the α(Al) + β(Si) eutectic, and the Mg_2_Si phase, contained in the α(Al) + Mg_2_Si + β(Si) eutectic, the β-Al_5_FeSi phase was identified. Due to its lamellar-needle morphology and so-called “needle tip phase” and lengths of up to 1000 µm (during gravity casting), the β-Fe phase separations are brittle and are preferred sites for stress propagation. It has a single-stranded structure with lattice parameters a = b = 0.16 nm and c = 4.14 nm and an average chemical composition of about 55–63 wt.% Al; about 26 wt.% Fe and about 13.0–15.2 wt.% Si [22]. The preferred direction of growth in one direction causes the phase β-Al_5_FeSi (especially at maximum iron content) to be cracked [57], exerting an unfavorable influence on the mechanical properties of Al-Si(Fe) alloy castings. The proportion of this phase and its size depend on the iron content, as shown in Figure 9.

After adding manganese, the α-Al_15_(Fe,Mn)_3_Si_2_ phase with dendritic morphology was identified in the alloy microstructure (Figure 5 and Figure 7). As indicated in the introduction, this phase replaced the β-Al_5_FeSi phase due to the similarity in the type of iron and manganese crystals. The results reported in [50] show that the four-component Al_15_(Fe,Mn)_3_Si_2_ phase is a three-component Al_15_Mn_3_Si_2_ phase, in which iron atoms have replaced up to 90% of the manganese atoms. The stoichiometry of the Al_15_(Fe,Mn)_3_Si_2_ phase is 0–31 wt.% Fe, 2–29 wt.% Mn, and 8–13 wt.% Si, while the Al_15_Mn_3_Si_2_ phase is a cubic crystal whose lattice parameter changes from a = 12.65·10^−10^ m for 0 wt.% Fe to a = 12.5·10^−10^ m for 30 wt.% Fe [50]. The other components of the AlSi10MnMg alloy structure are unchanged, regardless of the proportion of scrap (iron content), as confirmed by studies [58,59,60,61].

**Figure 10:**
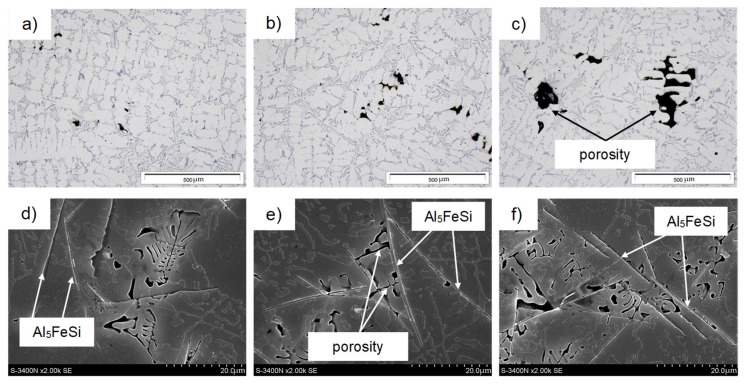
SEM microstructure of AlSi10MnMg alloy without manganese addition showing clusters of porosity with scrap content (**a**–**d**) about 50% (0.32 wt.% Fe); (**b**–**e**) about 65% (0.5 wt.% Fe); (**c**–**f**) about 70% (0.7 wt.% Fe); (**a**–**c**)—light microscope; (**d**–**f**)—SEM.

Studies of the microstructure of the AlSi10MnMg alloy also showed that with increasing iron content, or rather, iron phases, shrinkage-type porosity increases, as shown in Figure 10.

It can be seen from Figure 10e,f that the porosity is mainly concentrated between the long, needle-like separations in the β-Al_5_FeSi phase, thus reducing the interdendritic permeability and, therefore, blocking (inhibiting) the free flow of the liquid melt during cooling. During die casting, the moving front of crystallization “cannot keep up” with filling with liquid metal the places “closed” by the β-Fe phases, causing the formation of pores and shrinkage micropatterns in their vicinity. Thus, it is clear that β-Al_5_FeSi phases (especially when they crystallize originally) hinder the free access to fill the α(Al) interdendritic areas, causing high shrinkage porosity. This is consistent with Taylor’s findings [51,62,63].

Campbell presents a different approach to the causes of porosity in aluminum alloys [24,64,65], indicating that the primary source of porosity is the oxides formed when the alloy is transferred from the furnace to a ladle or mold. However, it should be noted that the AlSi10MnMg alloy was not transferred from the furnace to the ladle. Aluminum alloys are prone to oxide formation (liquid aluminum has a high affinity for oxygen), and oxidation occurs in milliseconds when the aluminum surface is in contact with atmospheric oxygen and moisture. However, the alloy under study (in the liquid state) was not exposed to gas entry, so forming “bifilms” in it is rather unlikely. On this basis, it can be concluded that although the contribution of oxide “bifilms” to the formation of porosity in aluminum alloys is possible using refining and protective coatings, they do not have a significant effect on the formation of porosity.

**Figure 11:**
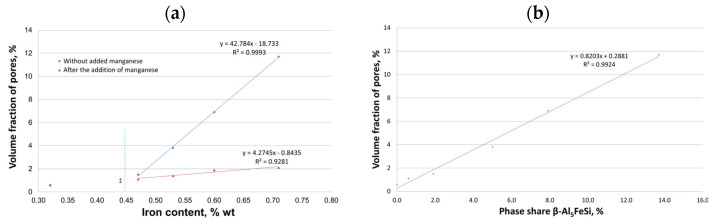
Effect of iron content on (**a**) percentage of porosity without and with manganese addition; (**b**) β-Al_5_FeSi phase on porosity in the AlSi10MnMg alloy without manganese addition.

The presence of voids affects the weakening of the material, causing local stresses first and then microcracks, which transform into cracks during the operation of Al-Si(Fe) alloy castings. This is because long, needle-like separations of β-Fe phases oriented with their longer dimension perpendicular to the given load undergo decohesion. In contrast, those arranged in parallel—cracks along their arms undergo fragmentation [57]. Thus, it should be concluded that β-Al_5_FeSi phases, which appear in the microstructure of AlSi10MnMg alloy especially after exceeding the content of about 0.7 wt.% Fe, constitute the germs of pores and shrinkage microparticles, causing an increase in porosity in castings and a decrease in the feeding capacity by reducing the flow of liquid melt between the branches of the dendrites of the α(Al) solid solution. They provide sites for the initiation of microcracks, thus reducing strength properties, especially ductility, determined by yield strength and elongation (Figure 4c,d). The effect of iron content and β-Al_5_FeSi phase on porosity in AlSi10MnMg alloy is shown in Figure 11.

Figure 11a shows that in AlSi10MnMg alloys without manganese addition, the proportion of porosity increases very rapidly according to the regression-line equation y = 4.2745x − 0.8435, while without manganese the increase is much smaller: y = 42.784x − 18.733.

## 4. Summary and Conclusions

During the crystallization of Al-Si-Mn-Mg and Al-Si-Mn-Mg(Fe) alloys, many phases are formed. Still, sometimes, the accompanying exothermic reactions are so minor that they are difficult to record by thermal analysis. According to Mondolfo [61], the first phase to form in the Al-Si-Fe-Mn system (in many industrial Al-Si alloys with manganese and iron) is Al_6_(Fe,Mn). However, this phase reacts peripatetically with the liquid to form the Al_15_(Fe,Mn)_3_Si_2_ phase or does not separate if there is no manganese in the alloy. Hence, the cooling curve of the AlSi10MnMg alloy lacks a reaction originating from its separation. Thus, crystallization of the AlSi10MnMg alloy selected for this study (using the ATD method) probably occurs according to the following sequence:

- To a content of about 0.45 wt.% Fe:
  (3) Liq. → α(Al) + α(Al) + β(Si) + α(Al) + (Al;Si;Fe) + β(Si) + α(Al) + (Mg_2_Si) + β(Si)
  (these eutectics can crystallize simultaneously);
- With a content of about 0.45 to 0.7 wt.% Fe:
  (4) Liq. → α(Al) + Al5FeSi + α(Al) + β(Si) + α(Al) + (Mg_2_Si) + β(Si)
  (crystallization of eutectics as above);
- With a content of more than 0.7 wt.% Fe:
  (5) Liq. → Al_5_FeSi + α(Al) + α(Al) + β(Si) + α(Al) + (Mg_2_Si) + β(Si)
  (crystallization of eutectics as above).

On the other hand, after the introduction of manganese (Mn/Fe = 1/2), the crystallization of AlSi10MnMg alloy occurs according to the following order:

(6) Liq. + Al_5_FeSi → α(Al) + α(Al) + β(Si) + α(Al) + (Mg_2_Si) + β(Si) + Al_15_(Fe,Mn)_3_Si_2_

(eutectics can crystallize simultaneously)

The order of the crystallizing phases is consistent with the results of [63]. As a result of reaction (6), unfavorable lamellar-igneous separations of Al_5_FeSi transform into Al_15_(Fe,Mn)_3_Si_2_ phase with dendritic morphology (sometimes so-called “chinese script”). The result of this reaction is a change in the mechanical properties of the AlSi10MnMg alloy with an increasing proportion of circulating scrap (from 50 to 70% by weight of the charge), i.e.:

- Improvement in HB hardness by about 37%;
- Reduction in UTS tensile strength by about 16%;
- Reduction in YS yield strength by about 16%;
- Slight reduction in elongation of about 18% compared to an alloy with 50% scrap (0.32 wt.% Fe content).

Based on the study of AlSi10MnMg alloy with an increasing proportion of circulating scrap (increasing iron content), the following conclusions were made:

1. The alloy’s crystallization (under gravitational conditions) with a content of up to 0.45 wt.% Fe proceeds traditionally, as for silumin, which has a slightly sub-eutectic composition with manganese and magnesium content. At contents of about 0.45 to 0.7 wt.% Fe, a change in the order of crystallization takes place—the Al_5_FeSi phase separates pre-eutectically if the iron content exceeds about 0.7 wt.%—the Al_5_FeSi phase crystallizes originally. This phase does not affect the crystallization of the other components, i.e., dendrites α(Al), eutectics α(Al) + β(Si), and eutectics containing the Mg_2_Si phase.
2. After adding AlMn20 master alloy, the Al_15_(Fe,Mn)_3_Si_2_ phase was identified, whose crystallization occurs in the temperature range of 540–555 °C and increases slightly with increasing manganese addition.
3. When introducing manganese into the alloy, the quotient Mn/Fe of 1/2 must be observed. A higher manganese content results in the formation of sludge particles, which are deposited on the surface of the liquid alloy and can adversely affect its mechanical properties.
4. Increasing the scrap content of the AlSi10MnMg alloy decreases UTS, YS, and elongation values, hence the need for manganese addition. This transformation transforms the morphologically unfavorable β-Al_5_FeSi phase into α-Al_15_(Fe, Mn)_3_Si_2_ with dendritic morphology.
5. Without the addition of manganese, the results of mechanical properties are characterized by a large scatter (measured by standard deviation). In contrast, after the introduction of manganese, the scatter is much smaller, indicating a more homogeneous alloy structure.
6. Up to a content of about 0.45 wt.% Fe, there was no effect of iron on shrinkage porosity. Only after this value is exceeded is there a correlation between the amount of Al5FeSi phase precipitates and porosity.

The main conclusion is that increasing the proportion of circulating scrap in the AlSi10MnMg alloy for gravity casting is possible but only with a manganese-to-iron addition ratio of roughly 1:2.

The results presented here are only the beginning of a broader research program on the effect of increased scrap (iron content) on the functional properties of Al-Si-Me (Me = Cu; Mg; Mn; Ni) alloys of sub-, peri- and super-eutectic composition, and were intended to show general trends in the change in the mechanical properties studied. To further evaluate the effect of increased iron content on other performance characteristics of aluminum alloys, further studies are planned:

- The effect of casting process parameters (e.g., mold temperature, pouring speed) on the morphology and distribution of the β-Al_5_FeSi phase in castings with an iron content of more than 0.7 wt.% will be examined. It is known that in die-cast Al-Si-Mn(Fe) alloys, the allowable iron content is much higher (from about 1.0 to 2.0 wt.%) compared to gravity castings (from 0.4 to about 0.7 wt.%).
- Heat flow and solidification, pore formation, and phase transformation processes will be simulated to ensure the best possible metallurgical quality of the alloys studied.
- The effect of heat treatment on the properties of alloys with different manganese content will be investigated, especially in terms of aging enhancement and grain refinement of casting alloys.
- Long-term performance of AlSiMnMg alloys will be evaluated under extreme conditions (e.g., for different operating temperatures; the influence of iron and manganese on corrosion; and the possibility of carrying out thermo-chemical treatment of the surface of castings made of Al-Si-Me (Me=Cu; Mg; Mn; Ni) alloys with an increased proportion of scrap).

## Figures and Tables

**Table 1:** Results of the chemical composition of the AlSi10MnMg alloy (arithmetic mean).

Sample No.	Element Content, wt.% ^1^									
	Si	Fe	Mn	Mg	Cu	Cr	Zn	Ti	Sr	Other ^2^
	Without Added Manganese									
1	9.64	0.32	0.18	0.49	0.03	<0.01	0.014	0.059	0.015	<0.02
2	9.88	0.44	0.20	0.52	0.03	<0.01	0.012	0.062	0.014	<0.02
3	9.97	0.48	0.22	0.48	0.02	<0.01	0.014	0.058	0.014	<0.02
4	9.91	0.51	0.21	0.52	0.04	<0.01	0.013	0.061	0.013	<0.02
5	9.76	0.60	0.21	0.54	0.04	<0.01	0.014	0.064	0.014	<0.02
6	9.69	0.71	0.19	0.54	0.03	<0.01	0.013	0.060	0.013	<0.02
After the addition of manganese (AlMn)										
1Mn.	9.58	0.32	0.18	0.44	0.02	<0.01	0.013	0.053	0.014	<0.02
2Mn.	9.92	0.43	0.22	0.48	0.02	<0.01	0.013	0.060	0.014	<0.02
3Mn.	9.77	0.47	0.25	0.46	0.02	<0.01	0.012	0.060	0.013	<0.02
4Mn.	9.90	0.53	0.25	0.50	0.03	<0.01	0.014	0.058	0.014	<0.02
5Mn.	9.56	0.58	0.30	0.49	0.03	<0.01	0.011	0.061	0.013	<0.02
6Mn.	9.83	0.70	0.36	0.50	0.02	<0.01	0.011	0.061	0.015	<0.02

^1^—the rest of aluminum; ^2^—e.g., nickel, lead, tin.

**Table 2:** Characteristic crystallization temperatures and parameters of AlSi10MnMg alloy from ATD analysis ^1^.

Sample No.	Scrap Content, %	Characteristic Crystallization Temperatures and Parameters ^2^, °C								
		Point X T_E(Fe)_	Point A T_liq(α)_	Point B T_Emin(α+β)_	Point C T_E(α+β)_	Point Y T_E(AlFeMn)_	Point D T_E(Mg)_	Point E T_sol._	Eutectic Recalescence α + β T_Emin._ − T_E(α+β)_	Crystallization Range T_liq._ − T_sol._
**Without added manganese**										
1	50	----	565	556	560	----	542	529	6	36
2	55	----	566	556	561	----	543	530	5	36
3	60	----	568	555	560	----	542	530	5	38
4	65	564	568	556	560	----	544	530	6	38
5	70	637	569	558	564	----	543	529	6	40
6	75	661	570	560	566	----	545	528	6	52
**After the addition of manganese (AlMn20)**										
1Mn.	50	----	569	555	561	540	436	526	6	43
2Mn.	55	----	571	556	561	543	539	525	5	46
3Mn.	60	----	570	555	560	547	539	527	5	43
4Mn.	65	----	569	557	563	550	540	530	6	39
5Mn.	70	----	570	556	562	551	538	522	6	48
6Mn.	75	----	568	556	562	555	540	521	6	47

^1^—the symbols in the table are the same as in Figure 2; ^2^—values rounded to integer.

## Data Availability

The original contributions presented in this study are included in the article. Further inquiries can be directed to the corresponding author.
